# Personalised PEEP that yields the highest lung compliance versus optimal balance between overdistension and collapse during PSV: authors’ reply to Dr Stenqvist

**DOI:** 10.1186/s13054-022-04261-0

**Published:** 2022-12-09

**Authors:** Elena Spinelli, Douglas Slobod, Tommaso Mauri

**Affiliations:** 1 Department of Anesthesia, Critical Care and Emergency, Fondazione Istituto di Ricovero e Cura a Carattere Scientifico Ca’ Granda, Ospedale Maggiore Policlinico, Via F. Sforza 35, 20122 Milan, Italy; 2 Department of Critical Care Medicine, McGill University, Montreal, QC Canada; 3 Department of Pathophysiology and Transplantation, University of Milan, Milan, Italy

## Dear Editor,

We recently published a novel method to personalize PEEP in hypoxemic patients undergoing pressure support ventilation [1]. We take the opportunity to reply to Dr. Stenqvist’s comments to further clarify the details of our approach.

The method we presented in the brief report integrates electrical impedance tomography and transpulmonary pressure (P_L_) monitoring to identify optimal PEEP in intubated patients on pressure support ventilation (PSV). Given the well-known side effects of deep sedation and the dramatic impact of controlled ventilation on the risk of muscular atrophy [2], switching to assisted ventilation early is often pursued in intubated patients with AHRF. During assisted ventilation, titration of ventilation settings aiming for lung protection needs to consider an elusive variable: the patient’s inspiratory effort. Inspiratory effort adds to airway driving pressure to generate transpulmonary driving pressure (ΔP_L_), and thus to lung stress. For a given airway driving pressure, ΔP_L_ can vary substantially depending on patient effort. In clinical practice, P_L_ is calculated as the difference between airway pressure and esophageal pressure and ΔP_L_ as the difference in P_L_ between end-inspiration and end-expiration.

Patient effort, and thus its contribution to ΔP_L,_ can be affected by PEEP. The impact of PEEP on effort depends on factors including changes in lung compliance (balance between recruitment and overdistension) [3] and gas exchange, but it could also be affected by changes in neuro-ventilatory efficiency due to PEEP-induced modifications in the conformation of the diaphragm [4].

By integrating dynamic monitoring of ΔP_L_ through the use of esophageal pressure, we performed a PEEP trial that accounted for the impact of PEEP on inspiratory effort in three hypoxemic patients on PSV. In all cases, the identified PEEP that balanced the percentage of alveolar collapse and overdistension corresponded to the step with the highest lung compliance, suggesting that the PEEP-induced change in lung mechanics is a main determinant of the effect of PEEP on effort.

In contrast with our approach, the method proposed by Dr. Stenqvist uses a calculation of ΔP_L_ from lung compliance indirectly derived from measurements of end-expiratory lung volume at two PEEP levels under passive conditions. Then, a lung pressure–volume curve can be constructed to identify the PEEP associated with best lung compliance. However, measures performed during passive ventilation do not always coincide with respiratory mechanics assessed during PSV (e.g., dorsal recruitment can improve regional lung compliance). In addition, given the very small sample size that we studied, we cannot conclude that optimal EIT-ΔP_L_-based PEEP always corresponds with the PEEP that yields the highest lung compliance. Until more patients are studied, a more complete assessment that accounts for changes in inspiratory effort at each step should be adopted.

## Data Availability

Not applicable.
